# Exploration of IMDC model in patients with metastatic renal cell carcinoma using targeted agents: a meta-analysis

**DOI:** 10.1590/S1677-5538.IBJU.2019.0423

**Published:** 2020-02-20

**Authors:** Guiya Jiang, Shuqiu Chen, Ming Chen

**Affiliations:** 1 Southeast University School of Medicine China School of Medicine, Southeast University, China; 2 Southeast University Department of Urology Zhongda Hospital NanJing China Department of Urology, Southeast University, Zhongda Hospital, NanJing, 210009, China

**Keywords:** Carcinoma, Renal Cell, Meta-Analysis [Publication Type], Prognosis

## Abstract

**Purpose:**

To explore the International Metastatic Renal Cell Carcinoma Database Consortium (IMDC) model application for predicting outcome of patients with metastatic renal cell carcinoma using targeted agents.

**Materials and Methods:**

We performed a literature review of 989 articles. The selecting process used preferred reporting items for systematic reviews and meta-analyses (PRISMA). All included studies were assessed by Newcastle-Ottawa scale. Results of individual studies were pooled using Stata 14.0 software.

**Results:**

A total of 17 articles were included. Most articles provided univariate and multivariate analysis of IMDC model prognosis. Combined HRs were 1.58 (95% CI 1.34-1.82) and 3.74 (95% CI 2.67-4.81) for univariate PFS of intermediate to favorable and poor to favorable respectively. In the category of multivariate PFS, combined HRs were 1.27 (95% CI 0.99-1.56) and 2.29 (95% CI 1.65-2.93) with intermediate to favorable and poor to favorable respectively. Regarding univariate OS, combined HRs were 1.93 (95% CI 1.62-2.24) and 6.25 (95% CI 4.18-8.31) with intermediate to favorable and poor to favorable respectively. With multivariate OS, combined HRs were 1.32 (95%CI 1.04-1.59) and 2.35 (95%CI 1.69-3.01) with intermediate to favorable and poor to favorable respectively.

**Conclusion:**

In summary, analysis of currently available clinical evidence indicated that IMDC model could be applied to classify patients with metastatic renal cell carcinoma using targeted agents. However, different types of targeted agents and various areas could affect the accuracy of the model. There was also a difference in predicting patients' PFS and OS.

## INTRODUCTION

Renal cell carcinoma (RCC) represents approximately 3% of all cancers, with the highest incidence occurring in western countries. Generally, during the last two decades, there has been an annual increase of 2% in incidence both worldwide and in Europe, leading to approximately 99, 200 new RCC cases and 39.100 kidney cancer-related deaths within the European Union in 2018 (1). According to the 2019 tumor statistics, there were 44.120 new kidney cancer men and 29.700 women in the United States, with the incidence rates being third and eighth respectively (2). Although most RCC cases are diagnosed at an early stage, approximately 20% of patients undergoing curative nephrectomy will subsequently develop metastasis during the follow-up period (3). Many new therapeutic drugs have emerged, such as immune checkpoint drugs based on PD-1/PD-L1 or CTLA4 as representative drugs, targeted agents are still the mainstream drugs for the treatment of metastatic renal cell carcinoma. Because of the poor prognosis of metastatic renal cell carcinoma, it is important to choose appropriate prognostic factors for communication with patients and their families, to determine treatment options, and to group people in clinical trials. The most widely used prognostic models for the prognosis of metastatic renal cancer is International Metastatic Renal Cell Carcinoma Database Consortium (IMDC) model (4). IMDC model was based on prognostic data from populations treated with various targeted drugs (5). Although the applicability of the model has been verified by some articles like Kwon's article (6), there are also articles like Peltola's (7) article that provide different conclusions. Therefore, we conducted this study to explore the IMDC model application for predicting outcome in patients with metastatic renal cell carcinoma using targeted agents.

## MATERIALS AND METHODS

### Search strategy

**Figure 1:**
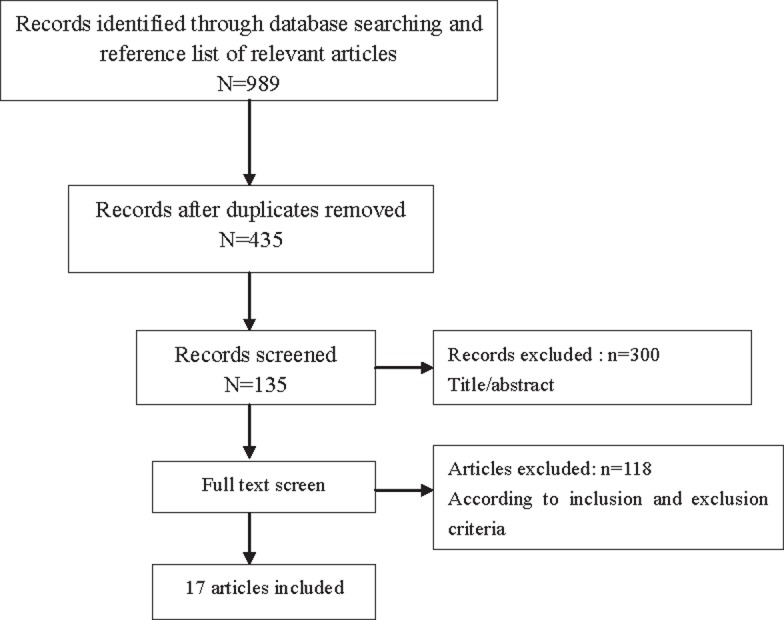
Selective process using preferred reporting items for systematic reviews and meta-analyses (PRISMA) statement.

We performed a literature review of articles published before June 31, 2019 using the PubMed, Web of Sciences and Embase Databases. The main search terms used were “metastatic renal carcinoma”, “prognosis”, “TKI”, “mTORi”, “sunitinib”, “sorafenib”, “pazopanib”, “axitinib”, “bevacizumab”, “everolimus”, “temsirolimus” et al. and their combinations. Additional references were identified from the reference list of each article. Two reviewers carried out this process independently. The selecting process using preferred reporting items for systematic reviews and meta-analyses (PRISMA) (8) statement was exhibited in Figure-1 following the inclusion and exclusion criteria.

### Inclusion and Exclusion Criteria

Inclusion criteria: (1) patients were confirmed with metastatic renal carcinoma pathologically, (2) used targeted agents, (3) provided survival outcome based on IMDC model such as progression-free survival (PFS) or overall survival (OS) with hazard ratio (HR) and 95% confidence intervals (95% CI).

Exclusion criteria: (1) cohort of patients including other therapy like cytokine or immune checkpoint drugs, (2) articles providing data from the same population, (3) not in English.

### Data synthesis and analysis

All included studies were assessed by New-castle-Ottawa scale which provided a score from a possible total of nine scores. Key quality areas assessed included: (1) selection of study groups, (2) comparability of the groups, and (3) assessment of the outcome. High scores indicated high quality, a study with a score ≥6 was regarded as high quality, while a score <6 was regarded as moderate or low quality (9). Results of individual studies were pooled using Stata 14.0 software (Stat Corp, College Station, TX, USA). Meta-analytical method was inverse variance method. We used the I^2^ statistic test to assess the heterogeneity between studies. I^2^ ranges are from 0% to 100% (a value of 0% represents no heterogeneity, 0% <I^2^ <25% represents mild, 25% ≤I^2^ <50% represents moderate, 75% ≤I^2^ represents great heterogeneity). When I^2^ <50% or P_heterogeneity_ >0.1, no obvious heterogeneity existed among the studies. To achieve a relatively conservative conclusion, the random-effects (RE) model was applied (10, 11). Publication bias was assessed using a funnel plot and Egger's test. Sensitivity analysis was used to estimate the robustness of pooled results. P value <0.05 was considered to be statistically significant difference in studies.

## RESULTS

### Characteristics of included studies

According to the search strategy, 989 articles were retrieved from the electronic databases. By excluding duplicate reports and screening the abstracts, 135 articles were read by full text. The remaining articles were further excluded upon full-text review for several reasons, such as a lack of sufficient data to estimate HRs or duplicate publication in repeated cohorts. Finally, 17 articles were included for meta-analysis and the summary characteristics of articles were obtained (Table-1). Some articles provided different data from similar cohort of patients. Most articles provided univariate and multivariate analysis of HRs involving different factors for PFS and OS and we exhibited pooled results respectively.

**Table 1:** The summary characteristics of 17 included articles.

Author	Year	Country	Drug	Lines	Patient	Period	Follow-up (months)
Keizman	2014	Israel/US	Suni	combined	278	2004/2/1-2013/3/31	55
Yao	2018	China	suni or sora	NA	231	2007-2017	NA
Peltola	2017	Finland	suni	first line	137	2006/10/18-2012/5/31	NA
Kawai	2015	Japan	suni	first line	46	2008/11/1-2013/7/1	21.2
Giorgi	2014	Italy	suni	first line	181	2006/2/1-2011/9/1	30.4
Auclin	2017	France	evero	no first line	124	2007/2/1-2014/11/1	NA
Cai	2017	China	suni or sora	first line	143	2006/3/1-2015/7/1	22
Benoit	2013	Belgium/France	suni	first line	200	2005/1/1-2012/10/1	67
Bamias	2014	Greece/France/Belgium	suni	first line	186	2005/10/1-2012/1/1	34.07
Xia	2017	China	suni or sora	first line	110	2005/3/1-2014/6/1	NA
Wang	2016	China	suni or sora	first line	111	2005/3/1-2014/6/1	19.7
Miyake	2016	Japan	suni	first line	50	2008/5/1-2013/7/1	20
Lin	2019	China	suni or sora	first line	108	2005/3/1-2014/6/1	23.35
Kwon	2013	Korea	suni or sora	first or second	106	2007/4/1-2012/7/1	NA
Lolli	2016	Italy	suni	first line	335	NA	49
You	2016	Korea	suni, sora, pazo, or temsi	first line	325	2006/11/1-2013/6/1	NA
Kim	2018	Korea	suni or pazo	first line	554	2012/1/1-2016/11/1	16.4

Author	ccRCC	nccRCC	Favorable	Intermediate	Poor	NOS	Outcome	Survival time(months)
Keizman	211	67	60	163	55	7	PFS/OS	9/22
Yao	199	32	38	153	40	7	OS	17.5
Peltola	105	15	29	74	31	7	PFS/OS	8.6/24.4
Kawai	46	0	2	26	18	6	PFS/OS	9.6/18.1
Giorgi	165	16	44	103	33	7	PFS/OS	11/25.5
Auclin	109	15	40	62	22	7	OS	12.9
Cai	136	7	62	59	22	6	PFS/OS	11/27
Benoit	200	0	13	55	33	7	PFS/OS	12/20
Bamias	173	12	33	83	35	6	OS	20.96
Xia	88	22	22	60	68	7	PFS/OS	9.8/23.5
Wang	89	22	23	60	28	6	PFS/OS	NA
Miyake	40	4	10	29	11	6	PFS/OS	8.9/23.5
Lin	87	21	23	58	27	7	PFS/OS	NA
Kwon	85	21	18	54	16	7	PFS/OS	12/17.8
Lolli	315	20	117	176	42	6	PFS/OS	14.2/32.7
You	293	31	81	179	65	7	PFS/OS	16.2/26.1
Kim	NA	NA	114	345	90	6	OS	36.2/40.5

**Suni =** sunitinib; **Sora =** sorafenib; **pazo =** pazopanib; **evero =** everolimus; **temsi =** temsirolimus

### Univariate PFS

There were 8 articles (12-19) including 1618 patients in this category, and Kawai's article (17) provided HR of poor to favorable only. Among these patients, 1454 were clear cell RCC and 163 were non clear cell RCC, favorable, intermediate and poor risk group has 401, 821, 336 patients respectively. Sunitinib was the most commonly used agent.

### Intermediate to favorable

**Figure 2:**
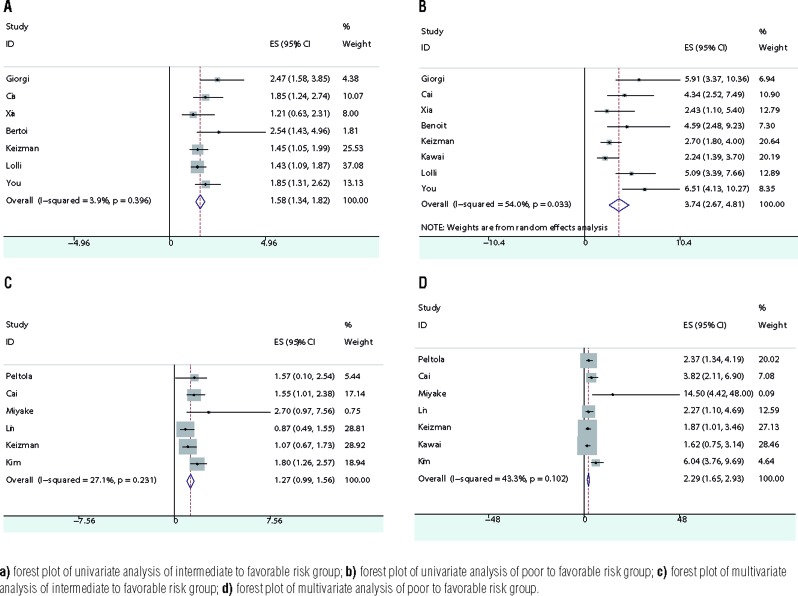
Combined HRs of IMDC model from PFS.

**Supplementary Figure 1:**
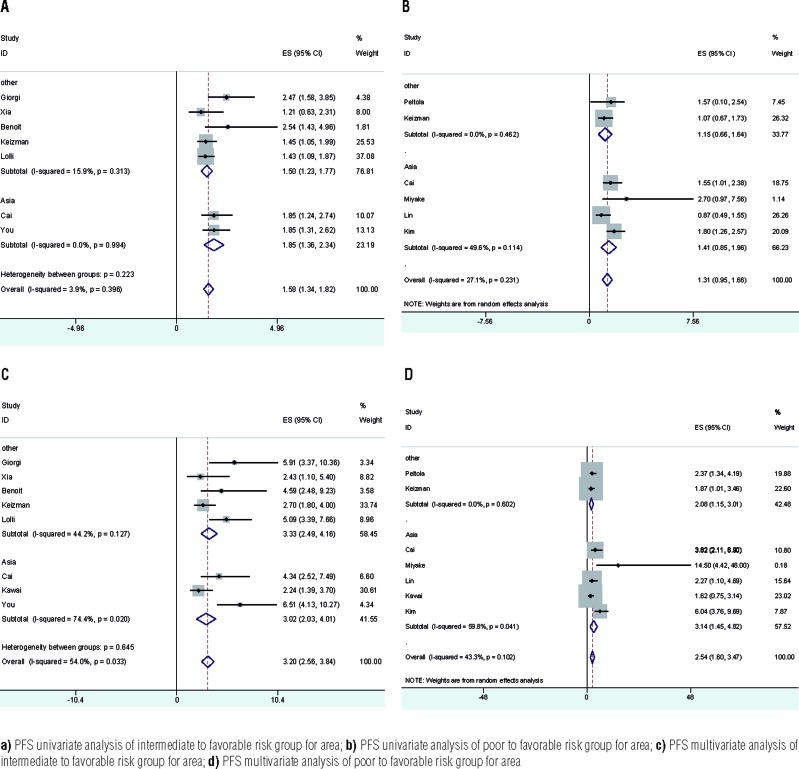
Subgroup analysis of area for PFS.

**Supplementary Figure 2:**
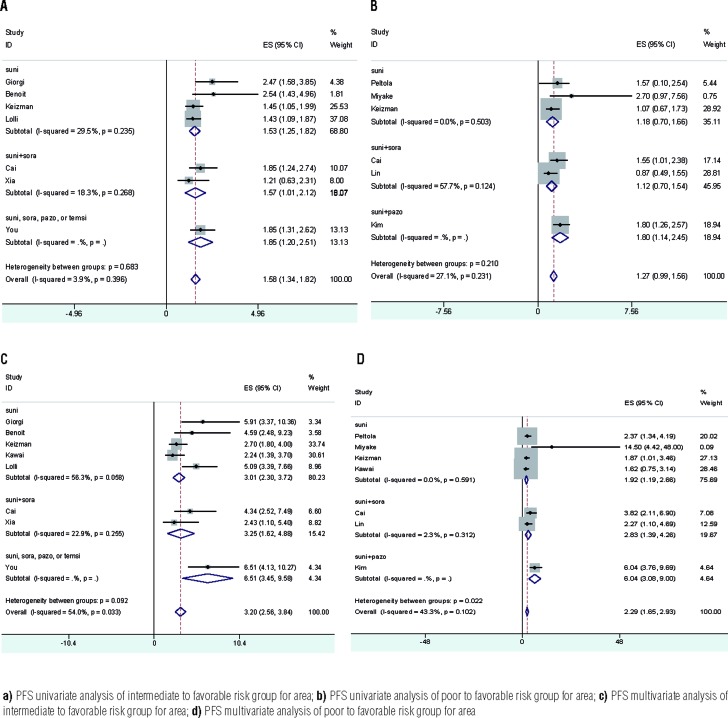
Subgroup analysis of drug type for PFS.

The combined HR was 1.58 (95% CI 1.34-1.82) and the forest plot is shown in Figure-2. According to funnel plot and egger's test (p=0.308), there was no publication bias. And sensitivity analysis showed the result was robust. Subgroup analysis showed the model was applicable in both Asia and other areas (Supplementary Figure-1). Whether the cohort of patients all took sunitinib alone or part of patients took sorafenib or pazopanib or temsirolimus, the model could effectively distinguish between favorable and intermediate-risk group (Supplementary Figure-2).

### Poor to favorable

The combined HR was 3.74 (95% CI 2.67-4.81) and the forest plot is shown in Figure-2. According to funnel plot and Egger's test (p=0.911), there was no publication bias. And sensitivity analysis showed the result was robust. Subgroup analysis showed the model was reliable in both Asia and other areas (Supplementary Figure-1). Whether the cohort of patients all took sunitinib alone or part of patients took sorafenib or pazopanib or temsirolimus, the model could separate patients between favorable and poor-risk group (Supplementary Figure-2).

### Multivariate PFS

There were 7 articles (7, 12, 16, 17, 19-21) I ncluding 1087 patients in the category, and Kawai's article (17) still provided HR of poor to favorable only. Among these patients, 918 were clear cell RCC and 145 were non clear cell RCC, favorable, intermediate and poor risk groups have 267, 588, and 229 patients respectively. Sunitinib was the most commonly used agent.

### Intermediate to favorable

The combined HR was 1.27 (95% CI 0.99-1.56) and the forest plot is shown in Figure-2. According to funnel plot and Egger's test (p=0.983), no publication bias was detected. And sensitivity analysis showed the result was not robust. When Lin and Keizman's article was omitted, the result changed to 1.43 (95% CI 1.09-1.77) and 1.35 (95% CI 1.01-1.69) respectively. Interestingly, only in Keizman's article the targeted agents were not used as first line therapy. Subgroup analysis showed the model was not applicable in both Asia and other areas (Supplementary Figure-1). Whether the cohort of patients all took sunitinib alone or part of patients took sorafenib or pazopanib or temsirolimus, the model was not efficient between favorable and intermediate-risk group (Supplementary Figure-2).

### Poor to favorable

The combined HR was 2.29 (95% CI 1.65-2.93) and the forest plot is shown in Figure-2. According to funnel plot and Egger's test (p=0.962), no publication bias was detected. And sensitivity analysis showed the result was robust. Sub group analysis showed the model was applicable in both Asia and other areas (Supplementary Figure-1). Whether the cohort of patients all took sunitinib alone or part of patients took sorafenib or pazopanib, the model was efficient to classify favorable and poor-risk group (Supplementary Figure-2).

### Univariate OS

In all 10 articles (6, 12-14, 16, 18, 18, 22-24) including 2419 patients in the category, among these patients, 1667 were clear cell RCC and 196 were non clear cell RCC. It was unfortunate that Kim's article did not provide specific number of patients with different pathological types. Favorable, intermediate and poor risk group had 565, 1227, and 419 patients respectively.

### Intermediate to favorable

**Figure 3:**
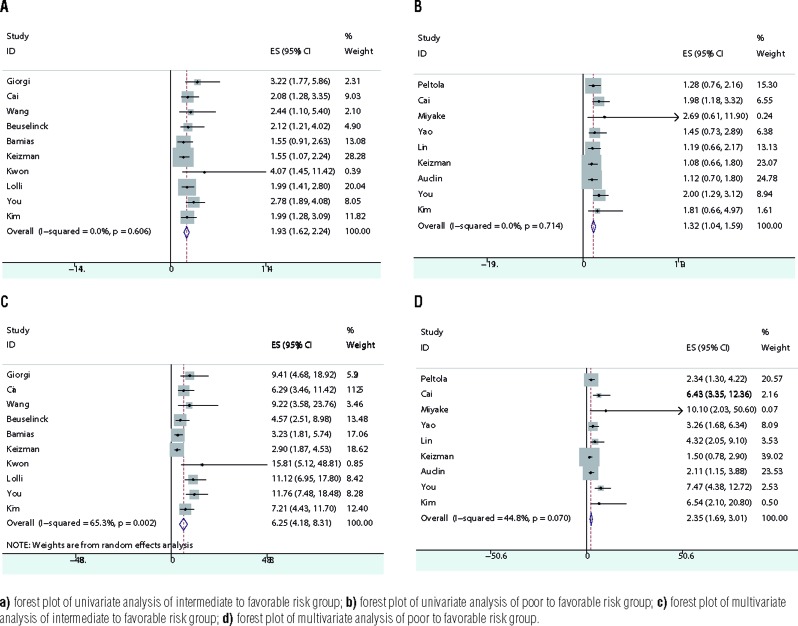
Combined HRs of IMDC model from OS.

**Supplementary Figure 3:**
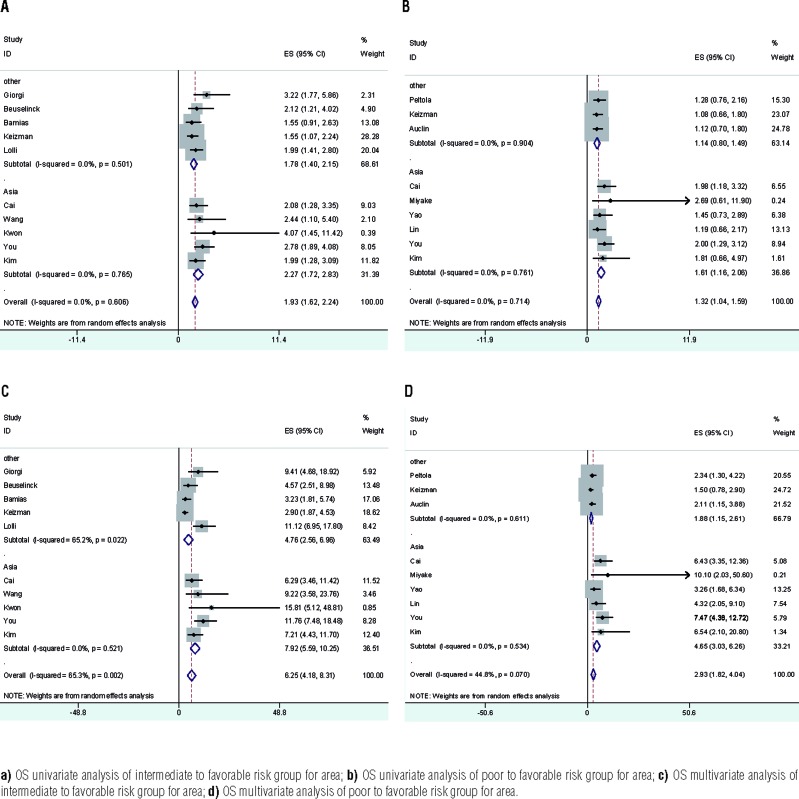
Subgroup analysis of area for OS.

**Supplementary Figure 4:**
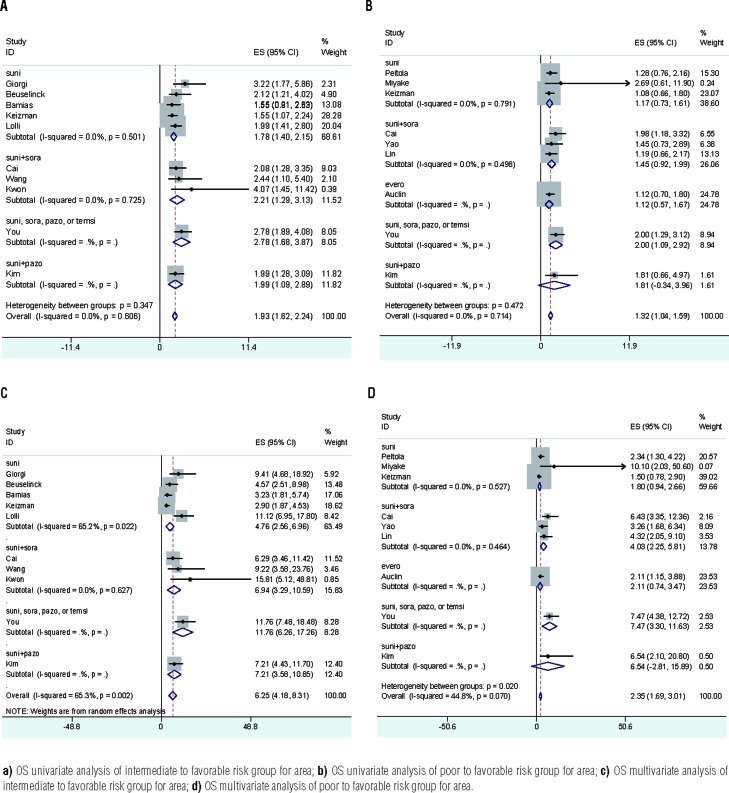
Subgroup analysis of drug type for OS.

The combined HR was 1.93 (95% CI 1.62-2.24) and the forest plot is shown in Figure-3. According to funnel plot and Egger's test (p=0.194), no publication bias was detected. Sensitivity analysis showed the result was robust. Subgroup analysis showed the model was applicable in both Asia and other areas (Supplementary Figure-3). Whether the cohort of patients all took sunitinib alone or part of patients took sorafenib or pazopanib or temsirolimus, the model was efficient to classify favorable and intermediate-risk group (Supplementary Figure-4).

### Poor to favorable

The combined HR was 6.25 (95% CI 4.18-8.31) and the forest plot is shown in Figure-3. According to funnel plot and Egger's test (p=0.596), no publication bias was detected. Sensitivity analysis showed the result was robust. Subgroup analysis showed the model was applicable in both Asia and other areas (Supplementary Figure-3). Whether the cohort of patients all took sunitinib alone or part of patients took sorafenib or pazopanib or temsirolimus, the model was efficient to classify favorable and poor-risk group (Supplementary Figure-4).

### Multivariate OS

A total of 9 articles (7, 12, 16, 19-22, 25, 26) including 1950 patients in the category, among these patients, 1180 were clear cell RCC and 192 were non clear cell RCC. Kim's article not providing specific number of patients with different pathological types was also included. Favorable, intermediate and poor risk groups had 457, 1122, and 363 patients, respectively.

### Intermediate to favorable

The combined HR was 1.32 (95% CI 1.04-1.59) and the forest plot is shown in Figure-3. According to funnel plot and Egger's test (p=0.551), no publication bias was detected. Sensitivity analysis showed the result was not robust. When Cai's article and You's article were omitted respectively, combined HR became not significant. Subgroup analysis showed the model was applicable in Asia. However, in other areas the model could not differentiate patients sufficiently (95% CI 0.80-1.49) (Supplementary Figure-3). Various types of targeted agents from cohort of patients affected the model's effectiveness to classify in favorable and intermediate-risk groups (Supplementary Figure-4).

### Poor to favorable

The combined HR was 2.35 (95% CI 1.69-3.01) and the forest plot is shown in Figure-3. According to funnel plot and Egger's test (p=0.555), no publication bias was detected. Sensitivity analysis showed the result was robust. Subgroup analysis showed the model was applicable in both Asia and other areas (Supplementary Figure-3). The model's efficiency was not reliable when it was applied to different types of targeted agents in the cohort of patients (Supplementary Figure-4).

## DISCUSSION

IMDC model including six independent factors such as KPS <80%, time from diagnosis to treatment <1 year; hemoglobin <LLN, Calcium >ULN, Neutrophils <ULN, and Platelets >ULN was first set in 2009 (5). After its occurrence, many studies applied it to make risk stratification of patients using targeted agents. However, there was not a systematic evaluation for the model. In-depth analysis of the existing literature was performed to explore the application of IMDC model. Interestingly, it was found that the model was also utilized to predict patient's PFS though it was first set to predict patient's overall survival. Actually, its application in predict patient's PFS had not been explored. This was the first study to validate their application in the area.

Most incorporated articles provide univariate and multivariate analysis of prognostic factors. For meta-analysis, univariate pooling can best reflect potential valuable prognostic factors despite the possibility of combining confounding factors leading to repetitive effects. Multivariate merging may be inherently heterogeneous due to the inconsistencies in the variables included in each article. Conversely, the statistically significant prognostic factors obtained through this combination may be able to withstand the challenges of different conditions and could be widely used.

According to our analysis, IMDC model was able to classify patients to different risk group with various PFS and OS except in the category of intermediate to poor risk group for PFS (95% CI 0.99-1.56). Simultaneously, the combined HR was larger in the category of univariate analysis than those in the category of multivariate analysis. It possibly suggested that IMDC model was affected by other existing factors. In other words, it should be taken into account when the model is incorporated as one independent prognostic factor to reform a new prognostic model. In addition, we also explored the applicability of this model in different drugs and different populations. There are a variety of targeted drugs, and we have included studies that simply use sunitinib as a treatment, as well as a combination of sorafenib, pazopanib, and even mTORi, such as temsirolimus. Based on the subgroup analysis, IMDC model was reliable on the univariate analysis of PFS and OS and multivariate analysis of PFS limited in the poor to favorable risk group. Its applicability was not stable in the category of multivariate analysis of PFS located in the intermediate to favorable risk group and multivariate analysis of OS. When it came to the area targeted agents were used, various results existed in different conditions. IMDC model was reliable on the univariate analysis of PFS and OS and multivariate analysis of PFS and OS limited in the poor to favorable risk group both in Asia and other areas. It was not reliable in the category of multivariate analysis of PFS located in the intermediate to favorable risk group both in Asia and other areas. However, it could be used in the multivariate analysis of OS in Asia not in other area. There were two main explanations for the difference. On one hand, unstable results were concentrated on the intermediate to favorable risk group, indicating the classification was not accurate enough. On the other hand, PFS results were more stable than OS results, indicating that OS was easier to be affected by other factors other than targeted drug therapy. There was no doubt that the number of studies included is an important factor affecting the outcome. More high-quality clinical studies could provide more robust results.

### Limitation and prospection

The findings of this systematic review should be considered in the context of the available evidence, which may be limited by selection bias and follow-up as reflected in the strength of evidence ratings. Due to there was not enough articles available, the application of the model for specific country or race was not explored. Meanwhile, most of the involved patients were ccRCC, the reliability of the model for nccRCC needed more studies to verify. Additionally, most articles used targeted agents as first line therapy except Keizman's, Auclin's and Kwon's articles (6, 16, 26), whether first line or second line of targeted therapy would influence the model was not explored. Although Heng's article (5) showed that there was no difference. And many other targeted agents such as axitinib were not covered in the included studies, leading to that the analysis was not particularly comprehensive. According to our analysis, the number of patients in the intermediate risk group was almost twice that of the other two groups, which was consistent with its initiative results (5). It indicated that a more specific subdivision could be made in the intermediate risk group.

## CONCLUSIONS

In summary, our analysis of currently available clinical evidence indicated that IMDC could be applied to classify patients with metastatic renal cell carcinoma using targeted agents. However, different types of targeted agents and various areas could affect the accuracy of the model. There was also a difference in predicting patients' PFS and OS. Based on the limitations of both the studies evaluated and our meta-analysis, further well-designed studies are needed to draw a more definite conclusion as to the clinical significance of IMDC model.
